# Chemical and Functional Characterization of a Novel European Black Soybean Variety

**DOI:** 10.3390/molecules31142417

**Published:** 2026-07-09

**Authors:** Marek Zdaniewicz, Szymon Lekowski, Barbara Mickowska, Stanisław Kowalski, Małgorzata Makarewicz

**Affiliations:** 1 Department of Fermentation Technology and Microbiology, Faculty of Food Technology, University of Agriculture in Krakow, Balicka Street 122, 30-149 Krakow, Poland; szymonlekowski@gmail.com (S.L.); malgorzata.makarewicz@urk.edu.pl (M.M.); 2 Centre for Innovation and Research on Prohealthy and Safe Food, University of Agriculture in Krakow, Balicka Street 104, 30-149 Krakow, Poland; 3 Department of Plant Products Technology and Food Hygiene, Faculty of Food Technology, University of Agriculture in Krakow, Balicka Street 122, 30-149 Krakow, Poland; barbara.mickowska@urk.edu.pl; 4 Department of Carbohydrate Technology and Cereal Processing, Faculty of Food Technology, University of Agriculture in Krakow, Balicka Street 122, 30-149 Krakow, Poland; st.kowalski@urk.edu.pl

**Keywords:** black soybeans, novel food proteins, chemical characterization, nutritional values

## Abstract

Interest in plant proteins and functional foods in the consumer diet is growing rapidly. One way to ensure food security is to diversify protein sources through the development of new plant varieties. This is of particular importance in the current era of climate change, when many historically cultivated varieties may be at risk. Due to their high protein content and favorable amino acid profile, soybeans have a wide range of nutritional and technological applications. The objective of this study was to asses a novel European black soybean variety that was obtained through phenotypic selection and stabilized across successive generations. The present study undertook a thorough evaluation of the chemical composition of the black soybean breeding line PLBPB1/24 and the commercial yellow soybean variety “Abelina”. The study, which employed analytical methods such as GC-FID, ICP-OES, ion-exchange chromatography, and spectrophotometric assays, revealed that PLBPB1/24 had a higher fat content, twice the amount of free amino acids (0.564 vs. 0.279 g/100 g), and an increased iron content (by 8%), while having a lower content of undesirable trypsin inhibitors (by 26%) compared to “Abelina”. These results confirm the potential of this new variety as a valuable source of nutrients, particularly in functional foods and plant-based diets.

## 1. Introduction

Soybeans (*Glycine max* (L.) Merr.) are one of the most important agricultural products worldwide. Among legumes alone, soybeans account for 75% of all crops [1]. In terms of production area, soybeans are second only to grains such as corn, rice, and wheat, making them one of the most widely cultivated non-grain crops [2]. The United States is the leading producer of soybeans, followed by Brazil, Argentina, India, China, Paraguay, Canada, and Ukraine. These countries collectively account for 92% of global production [3]. This plant is valued for its nutritional properties, including a high content (33–45%) of high-quality protein, essential fatty acids, and minerals, as well as numerous compounds that positively impact human health (e.g., isoflavones, tocopherols, and antioxidants) [4,5,6]. Protein quality depends not only on total protein content but also on amino acid composition, particularly the presence of essential amino acids that cannot be synthesized by the human body and must be supplied with food [7,8]. Adequate protein intake provides amino acids required for the metabolism of functional and structural proteins, including enzymes [9], while plant-based foods, especially legumes and soybeans, may also provide valuable amino acid profiles for meat-free diets [10,11,12,13]. Dietary protein requirements depend on physiological status and activity level and may range from basic recommendations to higher intakes in physically active individuals [14]. The lipid fraction is also nutritionally relevant because fats provide dietary energy, supply fatty acids, support the absorption of fat-soluble vitamins, and differ in their health effects depending on the proportions of saturated, monounsaturated, polyunsaturated, and trans fatty acids [15,16,17,18,19]. Soybean seeds are also an important source of macro- and microelements involved in regulatory, enzymatic, and structural functions, while potentially toxic elements should also be monitored in food materials [20,21,22,23,24,25,26,27]. Interestingly, 94% of soybeans are consumed as processed products such as tofu, soy sauce, and soy milk, while only a few percent are consumed as the seed itself [28]. This is linked to the growing consumption of soy products by vegetarians and vegans, who use them as substitutes for meat [29].

Soybeans can be distinguished by the color of their seed coat. There are yellow, green, brown, and black varieties [30]. Black soybeans, in particular, are widely distributed in Asia, where they have long been valued for their nutritional and medicinal properties [31]. Studies show that black soybeans have the highest antioxidant activity [32]. In addition to isoflavones, black soybeans contain other beneficial compounds: sterols, phytic acid, and phenols. One of the most significant classes of phenols present in black soybeans is that of anthocyanins, which are responsible for soybean coloration. Anthocyanins are a class of plant pigments whose color spectrum ranges from orange-red to dark blue-violet. They exhibit antioxidant, anti-inflammatory, and anticancer properties [33]. These compounds are responsible for the variation in polyphenol content observed between black soybeans and other varieties [31].

Despite their numerous beneficial properties, soybeans are also a source of certain anti-nutritional compounds. These compounds can affect human metabolism and inhibit the digestion and absorption of nutrients. One such compound is trypsin inhibitors, which impair protein absorption. Trypsin is a pancreatic serine protease involved in protein digestion, while soybean trypsin inhibitors, mainly Kunitz and Bowman–Birk inhibitors, reduce the activity of proteolytic enzymes such as trypsin and chymotrypsin [34,35,36,37]. This requires soybeans to be processed before consumption, which poses a major challenge for food manufacturers [34,38]. However, trypsin inhibitor activity can be reduced by thermal, physical, and biological processing methods, and its determination is important for assessing the quality of soybean seeds and soybean-based materials [38,39].

Although soybeans have been cultivated in Poland for about 140 years, it was not until the second decade of the 21st century that there was a significant increase in cultivated acreage. Nevertheless, soybean cultivation in Poland still accounts for only a small percentage of total domestic agricultural production. This is due to limited progress in breeding new varieties adapted to northeastern Europe’s climatic conditions [40]. Additionally, there has been no local variety of black soybeans on the Polish market until now. The present study aimed to evaluate the nutritional quality of the first Polish black soybean variety, which is currently undergoing registration. The majority of studies on this type of soybeans are of Asian origin and primarily focus on its antioxidant properties [30,31,41]. Therefore, the present study examined the content of proteins, fats, and minerals, as well as the presence of trypsin inhibitors, rather than focusing solely on antioxidant potential. This article represents the initial installment in a series of studies on a pioneering black-hulled soybean variety that has been developed in Europe. Anthocyanins, phenolic compounds, and antioxidant activity are the subject of a separate ongoing study, including the assessment of their changes under different processing conditions. Consequently, the present work expands existing knowledge on the differences between yellow and black soybeans, with particular emphasis on the nutritional and chemical parameters evaluated in this study.

## 2. Results and Discussion

### 2.1. Protein and Amino Acid Composition

The amino acid composition of soybean proteins from black and yellow soybean seeds is presented in Table 1. Free amino acids, which are not incorporated into proteins via peptide bonds and may be readily available, were also determined in this study (Table 2).

The overall protein content was higher in yellow soybeans (34.82 g/100 g) than in black soybeans (33.27 g/100 g). However, this variation is minimal (4.45%), and the amino acid composition of the protein is highly analogous in both varieties examined. Similar studies have shown that protein content, like that of other nutrients, depends heavily on variety and can vary even among variants with the same pod color. In their study, Lee et al. [42] analyzed several hundred varieties of black soybeans and determined the total protein content to be 39.7 g/100 g on average. This result is significantly higher than the result for the variety that was analyzed in this study. It is noteworthy, however, that this level was still lower than that of the yellow soybean variety (40.2 g/100 g), which served as the control. Choi et al. [43] also measured a higher protein content in black soybeans. After analyzing 24 varieties, they observed an average result close to 40 g/100 g. Additionally, the average result for the 10 yellow soybean varieties tested was slightly lower at 39.6 g/100 g. However, there are also studies in which the overall protein content in black soybeans was lower, even significantly lower, than in the Polish variety studied. Patel et al. [44] measured the protein content of local Indian varieties of black soybeans and found it to be 30 g/100 g for hulled seeds and 29.5 g/100 g for dehulled seeds. Chauhan et al. [45] investigated the effects of soaking and germination on changes in the nutrient composition of black soybeans. Prior to the implementation of these processes, the protein content was 24.67 g/100 g. The maximum level of protein recorded during the aforementioned processes was 27.59 g/100 g for the variant germinated for 72 h. The amino acid composition of the two varieties studied was very similar and consistent with other studies addressing this issue. Assefa et al. [46] examined the content of 9 essential amino acids in soybeans grown in 14 U.S. states, including tryptophan. In the present study, tryptophan was not determined because it is degraded during acid hydrolysis; therefore, it was excluded from the calculation. Consequently, the reported values represent the sum of the quantified essential amino acids, amounting to 12.44 and 13.07 g/100 g for PLBPB1/24 and “Abelina”, respectively, compared with the average value of 13.52 g/100 g reported by Assefa et al. Future studies should include a separate determination of tryptophan using an alkaline hydrolysis procedure followed by chromatographic analysis. Jaśkiewicz et al. [47] also determined the amino acid composition of protein in three soybean varieties, but their analysis was partial (they measured the concentration of only 12 amino acids). For most of them, the higher content was found in the black variety that was the subject of our study. The only exception was the concentration of methionine, which was higher in all three soybean varieties studied by these authors. Similar to a previously cited American study, the Polish team determined the presence of tryptophan, which was not assayed in the present study in either the black soybeans or the control sample. Although black soybeans had a slightly lower protein content than yellow soybeans, they had twice the amount of free amino acids (FAAs), which are not bound in peptide chains (0.564 vs. 0.279 g/100 g). However the measured free amino acids constituted only a small fraction of the total pool of protein-related compounds; their higher content represents an interesting compositional feature of the new variety. A particularly elevated level of two amino acids was observed: glutamic acid (0.101 g/100 g) and arginine (0.191 g/100 g). Arginine performs important functions, such as acting as a precursor in nitric oxide (NO) synthesis, and is essential for muscle growth, wound healing, and the repair of damaged tissues. Glutamic acid (glutamate) also plays a significant physiological role. It serves critical functions, particularly within the central nervous system and in numerous metabolic pathways. Moreover, glutamate is involved in the regulation of insulin secretion by the pancreas. Furthermore, FAAs have been demonstrated to facilitate the synthesis of antioxidants, hormones, and neurotransmitters, in addition to regulating cell growth [48]. Consequently, it is imperative to ensure that the body receives not only adequate protein but also the requisite amount of free amino acids. The black soybeans under study thus appear to be a suitable option, exhibiting a high protein content (not significantly different from the control variety) in conjunction with a substantially elevated concentration of FAAs.

### 2.2. Fat and Fatty Acid Profile 

In this study, the fat content and fatty acid profile of black and yellow soybean seeds were determined, and the results are presented in Table 3.

The results of the analyses for total fat content demonstrate that black soybeans exhibit a higher content (19.47 g/100 g) of this macronutrient in comparison to yellow soybeans (18.59 g/100 g). In addition, black soybeans have a higher percentage of saturated fatty acids in their fat composition: 25.19% compared to 22.48% in yellow soybeans. The percentage of individual saturated fatty acids, including palmitic and stearic acids, is presented in Table 3. Due to their higher saturated fatty acid content, black soybeans contain fewer monounsaturated and polyunsaturated fatty acids, such as oleic acid and omega-6 and omega-3 fatty acids. Black soybeans have a total content of unsaturated fatty acids of 74.66%. Yellow soybeans have a slightly higher content, at 77.52%. It should be kept in mind that the fat content and the proportions of individual fatty acids may vary significantly between varieties and depend on genetic differences as well as on growing conditions, climate and factors such as temperature during the growing season or rainfall [49,50]. As with saturated fatty acids, the exact proportions of unsaturated fatty acids are shown in Table 3. However, the ratio of omega-6 to omega-3 fatty acids is important when consuming polyunsaturated fatty acids; it should be approximately 5:1–4:1. This ratio minimizes the health risks associated with excessive polyunsaturated fatty acid (PUFA) consumption [51]. The ratio for black soybeans is 6.88:1. The difference in total unsaturated fatty acid content between the two varieties is mainly due to the higher linoleic acid content in yellow soybeans. Therefore, the omega-6 to omega-3 ratio in this variety is higher, at 6.99:1. According to Abdelghany et al. [52], the total fat content of soybeans typically ranges from about 10% to 22%. The researchers examined nearly 200 soybean varieties with different seed colors (brown, yellow, and black). Their study indicates that, on average, black soybeans have a lower fat content than brown and yellow soybeans. Conversely, other researchers studying black soybeans determined similar fat content levels to those obtained in our analyses. For example, Lee et al. [42] reported an average fat content of 18.6% across multiple varieties. Chauhan et al. [45] obtained a slightly lower but similar result of 16.36% total fat in the seeds. Thus, our measurements correlate with other studies but differ from those comparing yellow and black soybeans in the study by Abdelghany et al. [52]. The Polish variety that we studied generally had a higher fat content than the control variety with the typical yellow seeds. The findings of the present study for fatty acids deviate marginally from those of related studies. Shih et al. [53] presented the fatty acid profiles for two varieties of black soybeans and one variety of yellow soybeans. Their findings demonstrated that the varieties with dark-colored seeds contained fewer saturated fatty acids (palmitic and stearic acids) and more oleic acid. Higher oleic acid content is a nutritionally beneficial trait, which was also noted in our study. The content of this component was approximately 6.5% higher than in the control sample. In addition, similar to our study, Shih et al. [53] indicated that yellow soybeans contain higher amounts of omega-6 and omega-3 fatty acids. The SFA-to-UFA ratio was determined by Lee et al. [42], among other researchers. In their study, the results for the black and yellow soybeans were quite similar, standing at approximately 15:85. However, our results for this ratio differ significantly (25:75 for black soybeans and 22:78 for yellow soybeans). The study by Jaśkiewicz et al. [47] also shows significantly different proportions of saturated to unsaturated fatty acids. Their findings also demonstrate a lower proportion of saturated fatty acids in the overall profile. When analyzing an important aspect of the fatty acid profile, the omega-6 to omega-3 fatty acid ratio, it should be noted that this ratio was significantly different in all the previously cited studies [42,47,53]. A number of publications show a more favorable ratio of these two groups of acids for black soybeans, while others show it for yellow soybeans. This depends on the specific variety, just as the exact profile of the acids that make up the fats does. Minimal differences in the presence of individual fatty acids, even among unsaturated fatty acids, make it difficult to determine which soybean variety has a more favorable fat profile. However, it should be emphasized that, in the varieties we analyzed (black soybeans and the control variety), saturated fatty acid content is highest.

### 2.3. Macro- and Microelement Content

The mineral composition of the tested black and yellow soybean seeds is presented in Table 4.

The element present in the highest concentration in both analyzed soybean varieties is potassium (K), which has a higher content than all other macro- and micronutrients combined. The potassium content of the black soybean seeds was lower (15,275.5 mg/kg) than that of the control sample of yellow soybeans (16,436.7 mg/kg). The World Health Organization recommends a daily potassium intake of approximately 3500 mg [54]. The second most abundant element in soybeans is phosphorus (P), with contents of 6153.2 and 6136.6 mg/kg in the tested and control varieties, respectively. Magnesium (Mg) and calcium (Ca) follow. Moreover, the concentrations of cadmium and lead determined using this method were below the EU-permitted limit of 0.2 mg/kg [55]. In their study, Jaśkiewicz et al. [47] analyzed the composition of the most important macronutrients in three Polish varieties of yellow soybeans, obtaining the following average results: calcium—2553.3 mg/kg, phosphorus—6366.7 mg/kg, magnesium—2683.3 mg/kg, and potassium—16,466.7 mg/kg. These results are consistent with those obtained by our team. Additionally, we found that the calcium and magnesium content was higher in black soybeans than in the varieties studied by Jaśkiewicz et al. Further studies conducted in Poland (Medical University of Gdańsk) that focus on analyzing the macronutrient and micronutrient content of legume seeds (including soybeans) were carried out by Grembecka et al. [27]. In their study, the researchers used seeds purchased from local stores, though the soybeans were of Canadian origin. The black soybeans we evaluated are characterized by higher levels of essential macronutrients, with the exception of phosphorus, which was measured at an average level of 955 mg/100 g (approximately 9550 mg/kg). Additionally, they contain higher levels of important trace elements, such as iron and zinc, than the average values presented by the team from Medical University of Gdańsk. The difference is 57.2 mg/kg for iron and 38.4 mg/kg for zinc, both in favor of the pioneering black variety. When comparing our results to other studies on black soybeans, the work by Xiao et al. [56] is worth mentioning. The cited authors evaluated the effect of fermentation on the nutritional components of black soybeans. Both unfermented and fermented soybeans contain fewer essential elements than the Polish black soybeans described in the present study. An important point to note is the sodium content of black soybeans compared to the control sample. It is more than seven times higher in the former variety (92.8 vs. 12.4 mg/kg). The aforementioned study by Xiao et al. reports average sodium levels of 10.5 mg/kg and 22.4 mg/kg for unfermented and fermented soybeans, respectively, which are closer to the values determined in the control sample used in this study. Similarly, Grembecka et al. [27] determined the maximum sodium content of one sample to be 1.2 mg/100 g (12 mg/kg). However, literature data indicate that sodium content in soybean seeds may vary widely, with reported values ranging from 0.5 to 475 mg/kg [57], while mineral element content in plants may be influenced by both growing conditions and genetic factors [58]. From a nutritional perspective, according to WHO recommendations, daily sodium intake should be less than 2 g [59].

### 2.4. Trypsin Inhibitor Activity 

The trypsin inhibitor activity of PLBPB1/24 and the yellow reference variety “Abelina” is presented in Table 5.

The activity of antinutritional trypsin inhibitors in the black soybean variety was measured to be approx. 26% lower than in the yellow variety (30.22 and 40.71 TIU, respectively). These results are consistent with those of other researchers who have studied this issue, although direct comparisons between studies should be interpreted with caution because trypsin inhibitor activity may depend on the analytical method and unit used. For example, Kumar et al. [60] measured the trypsin inhibitor activity in 102 soybean genotypes grown in India, finding values ranging from 18.6 to 74.8 mg/g. The average was 45.9 mg/g. Due to the different units reported by the authors, the value was converted according to Pacheco et al. [61] (TIU/mg = mg/g × 1.9). Based on this conversion, 45.9 mg/g corresponds to a trypsin inhibitor activity of 87.21 TIU/mg. This converted value should therefore be considered only an approximate literature reference. This value is substantially higher than the activities determined in the present study for both soybean variants. Pesic et al. [62] conducted a similar study, analyzing 12 soybean varieties from two crops in Serbia. Their research team obtained an average TIA value of 86.2 TUI/mg. This result was approximately 2.9 times higher than that obtained for black soybeans and approximately 2.1 times higher than that obtained for the control sample and similar to the average result obtained in the previously cited study [60]. These studies are a suitable examples of the changes that have taken place in modern agriculture. Over the years, crop varieties with different nutritional and anti-nutritional profiles have been developed, and such direct comparisons may help illustrate this variability. When interpreting the results of this study in light of more recent research, the work of Meghana et al. [63] is worth referencing. They analyzed the nutrient content of 34 soybean varieties from eastern India. Their team measured an average trypsin inhibitor activity of 30.84 TIU/mg, a result very close to that obtained for the Polish black soybean variety (30.22 TIU/mg). However, it should be noted that the lowest value measured was 17.5 TIU/mg, and the highest result was 39.33 TIU/mg for one of the varieties. Like previous studies, this study shows that trypsin inhibitor content depends strictly on the variety, but modern varieties successfully reduce it. Alabi et al. [64] conducted an interesting comparative study in which they compared soybeans grown in Canada with Marama beans from Africa. The authors examined the effect of different thermal conditions on both species and their impact on trypsin inhibitor content. They measured an initial TIA of 66.98 TUI/mg for soybeans, while Marama beans exhibited significantly higher inhibitor activity, measured at 245.25 TUI/mg. The trypsin inhibitor content in soybean seeds is, of course, relevant to our study. Its concentration was higher than that of the varieties we examined: black and yellow soybeans. Although the lower trypsin inhibitor activity observed in PLBPB1/24 is a favorable nutritional feature, trypsin inhibitors are only one group of soybean anti-nutritional factors. Future studies should also include phytic acid, lectins, saponins, and mineral bioaccessibility, particularly because phytic acid may affect iron availability. Therefore, the higher iron content observed in PLBPB1/24 should be interpreted as an advantageous compositional characteristic rather than direct evidence of higher bioavailable iron.

## 3. Materials and Methods

### 3.1. Materials

The research material was a black soybean breeding line PLBPB1/24 (*Glycine max* (L.) Merr.), which—according to the authors’ and breeder’s knowledge—is the first black soybean variety developed in Poland and one of the first in Europe. It was developed through phenotypic selection conducted over successive plant generations by Sławomir Potrzebski in agriculture Sławomir, Małgorzata and Daniel Potrzebski (Bieńkowice, Poland). The selection was conducted over three consecutive growing seasons, from 2022 to 2024, and was based mainly on stable black seed coat color, seed uniformity, plant morphology, maturity, and yield potential. Phenotypic stability was assessed by observing the maintenance of these traits during successive propagation cycles. At the time of the study, the black soybean breeding line was undergoing the official registration process conducted by Research Centre for Cultivar Testing (COBORU).

The plant material used for chemical analyses originated from the 2024 growing season. The black soybean breeding line PLBPB1/24 and the commercial yellow soybean cultivar “Abelina”, used as the reference material, were cultivated side by side at the same location in Poland under comparable agronomic conditions and harvested at full seed maturity. After harvest, three independent seed samples were collected separately from the harvested seed material of each soybean genotype. Each sample was ground and homogenized independently and used for subsequent chemical analyses.

The tested line exhibited a high yield potential, estimated by the breeder to be approximately 20% higher than that of conventional yellow-seeded soybean varieties, despite being grown at a lower plant density. Furthermore, it demonstrated high phenotypic stability, consistently maintaining its characteristic black seed coat, seed morphology, plant architecture, and other distinctive agronomic traits across successive generations.

### 3.2. Analytical Determinations

#### 3.2.1. Amino Acid Composition, Free Amino Acids and Total Protein Content

##### Amino Acid Composition

Amino acid analysis was performed according to the method of Moore and Stein [65,66,67]. Lyophilized samples were hydrolyzed in liquid 6M HCl containing 0.5% phenol at 110 °C for 24 h under an argon atmosphere. The hydrolysates were lyophilized, dissolved in an appropriate volume of dilution buffer (sodium citrate buffer pH 2.2) and filtered through a 0.45 μm syringe filter (Millipore, Burlington, MA, USA) before being applied to the amino acid analyzer (Ingos, Prague, Czech Republic). Amino acids were determined by ion-exchange chromatography, with strong cation ion-exchanger and sodium-citrate elution buffer system followed by post-column derivatization with ninhydrin and spectrophotometric detection at 570 and 440 nm, according to standard protocol of the manufacturer [68]. Sulphur-containing amino acids are analyzed separately, as oxidation products obtained by performic acid oxidation, followed by standard hydrolysis procedure with HCl. For calibration of amino acid analyzer, the amino acid standard solution is used (Sigma, St. Louis, MO, USA). The method was performed according to a validated laboratory procedure. Validation included linearity, measurement range, LOD, LOQ, repeatability, intermediate precision, interlaboratory reproducibility, accuracy, and measurement uncertainty for individual amino acids. Evaluation of the acquired data was performed using the software of chromatographic device (CHROMuLAN v0.91, Pikron, Prague, Czech Republic). Tryptophan is not determined as it is destroyed during acid hydrolysis, and asparagine and glutamine turn to aspartic acid and glutamic acid, and these forms are determined.

##### Free Amino Acids

An amount of 0.1 g of sample was extracted twice with 0.75 mL of 0.1 M HCl. Deproteinization was performed by precipitation by sulfosalicylic acid (30 mg/mL). After centrifugation samples were filtered through a syringe filter and applied to chromatographic amino acid analyzer, analyses were performed according to standard protocol of manufacturer (Ingos, Czech Republic).

##### Total Protein Content

Total protein content was calculated as the sum of all amino acids determined during analysis of amino acid composition (it contains both protein and free amino acids).

#### 3.2.2. Fat and Fatty Acid Composition 

The fatty acid profile was determined as fatty acid methyl esters (FAMEs) after extraction of the fatty phase with ether and then after saponification and derivatization to fatty acid methyl esters [69]. The extracted fat (15 mg) was saponified using 0.5 mL of 0.5 N KOH (methanol solution) at 85 °C. Then, 1 mL of derivatizing solution (12% BF_3_ (methanol solution)) was added and the mixture was reheated at 85 °C. To isolate the fatty acid methanol esters, 1 mL of hexane and 5 mL of saturated NaCl solution were added to the sample. The organic layer containing fatty acid methyl esters was selected for analysis. Shimadzu GC 2010Plus gas chromatograph (Shimadzu Corp., Kyoto, Japan) with a flame ionization detector (FID) equipped with an SH-FAME column (30 m–0.32 mm–0.25 μm) was used to determine fatty acid profile. Operating parameters were as follows: FID detector temp., 250 °C; temperature dispenser, 220 °C; oven temp, 80 °C (held for 2.5 min), heated to 160 °C (60 °C/min) and held for 35 min; heated to 230 °C (15 °C/min) and held for 5 min; heated to 240 °C (5 °C/min) and held for 5.5 min. The carrier gas was helium (1.6 mL/min). The split ratio was 100. Individual fatty acid methyl esters were identified by comparison to the standard mixture of Supelco 37 com-ponent FAME Mix, Sigma-Aldrich Co. and of CLA isomers (Sigma-Aldrich Co., St. Louis, MO, USA). For each independently collected and homogenized seed sample, the analysis was performed in duplicate.

#### 3.2.3. Mineral Composition 

Mineral content analysis was performed using inductively coupled plasma atomic emission spectrometry with a multi-element ICP-OES analyzer (iCAP PRO XP Duo, Thermo Scientific™, Waltham, MA, USA), following pressure mineralization in a microwave digester.

Wet digestion was performed using a microwave digestion system (MARS Xpress, CEM Corporation, Matthews, NC, USA) in Teflon vessels using 65% Suprapur nitric acid (Merck, Darmstadt, Germany; catalog number 1.00441). The acid-to-sample ratio was 10 mL of nitric acid per 0.5 g of sample dry matter. The process was carried out with a maximum temperature of 200 °C. Quantitative determination was based on external multi-point calibration. Fresh calibration curves were prepared for each analytical series to ensure analytical accuracy and minimize instrumental drift. The following analytical wavelengths were used: Ca 393.366 nm, Mg 278.953 nm, K 766.480 nm, Na 589.592 nm, P 177.495 nm, Mn 257.610 nm, Fe 238.204 nm, and Zn 213.856 nm.

#### 3.2.4. Trypsin Inhibitor Activity Assay 

Trypsin inhibitor activity was determined using α-N-benzoyl-DL-arginine-p-nitroanilide hydrochloride (BAPA) as the substrate. Trypsin from bovine pancreas, Type I (Sigma-Aldrich/Sigma Life Science, St. Louis, MO, USA, product no. T8003; activity approx. 10,000 BAEE units/mg protein and 12,443 units/mg solid), was used to prepare the standard trypsin solution. Soybean meal (1 g) was extracted with 50 mL of 0.01 N NaOH by shaking for 1 h. The extract was centrifuged at 4000 rpm for 20 min, and the supernatant was collected. The extracts were diluted to obtain a level of inhibition corresponding to 40–60% of the activity of the standard trypsin solution. Aliquots of 0.8, 1.0, and 1.2 mL of the diluted extract were transferred into separate test tubes and adjusted to 2 mL with distilled water. Subsequently, 2 mL of trypsin solution was added to each tube. The mixtures were vortexed and incubated in a water bath at 37 °C for 5 min. After incubation, 5 mL of BAPA solution was added, and the reaction mixtures were further incubated for 10 min. The reaction was terminated by the addition of 1 mL of 30% acetic acid. Absorbance was measured at 410 nm using a UV–Vis spectrophotometer (UV-1900, Shimadzu, Kyoto, Japan). Distilled water was used as a blank. Trypsin inhibitor activity was expressed as trypsin inhibitor units per milligram of sample (TIU/mg). The results obtained for each extract volume were normalized to 1 mL of extract, and the final value was calculated as the mean of all determinations.

### 3.3. Statistics

Statistical analysis was performed using Statistica v. 10 software (StatSoft Inc., Krakow, Poland). For each soybean material, the results were calculated from three independently collected and separately homogenized seed samples. Analytical determinations were performed independently for each sample. The results are presented as mean values together with standard deviation (SD) and coefficient of variation (CV). The significance of differences between the black soybean breeding line PLBPB1/24 and the yellow soybean variety “Abelina” was evaluated separately for each parameter using Welch’s *t*-test. Differences were considered statistically significant at *p* < 0.05.

## 4. Conclusions

The black-seeded soybean breeding line PLBPB1/24 analyzed in this study showed promise as a variety with valuable nutritional properties. It has a similar, although slightly lower, protein content and a higher total fat content than the yellow soybean variety “Abelina”, which served as the control. It was also richer in free amino acids and showed approximately 26% lower anti-nutritional trypsin inhibitor activity, which may contribute to improved protein digestibility. Furthermore, PLBPB1/24 had a favorable macro- and micronutrient profile, including higher levels of important elements such as calcium, magnesium, and iron, while maintaining levels of other elements similar to those of the reference variety. Considering the results obtained in this study and comparing them with those of other studies, it can be concluded that this black soybean line, one of the first developed in Europe, has a high potential for large-scale cultivation.

## Figures and Tables

**Table 1:** Content of protein-building amino acids in black and yellow soybeans in g/100 g dry matter.

	Black Soybeans			Yellow Soybeans		
Amino Acid	Mean	SD	CV%	Mean	SD	CV%
Asp	3.99 ^a^	0.27	6.68	4.17 ^a^	0.23	5.52
**Thr**	1.28 ^a^	0.08	6.58	1.37 ^b^	0.08	5.84
Ser	1.73 ^a^	0.11	6.61	1.82 ^a^	0.1	5.49
Glu	6.63 ^a^	0.46	7.01	6.86 ^a^	0.38	5.54
Pro	1.71 ^a^	0.16	9.38	1.84 ^a^	0.15	8.15
Gly	1.44 ^a^	0.09	6.15	1.52 ^a^	0.08	5.26
Ala	1.47 ^a^	0.09	6.14	1.53 ^a^	0.08	5.23
**Val**	1.62 ^a^	0.1	6.41	1.7 ^a^	0.09	5.29
**Ile**	1.51 ^a^	0.1	6.55	1.59 ^a^	0.09	5.66
**Leu**	2.56 ^a^	0.17	6.49	2.71 ^b^	0.14	5.17
Tyr	1.27 ^a^	0.11	8.32	1.26 ^a^	0.1	7.94
**Phe**	1.68 ^a^	0.12	7.25	1.77 ^a^	0.12	6.78
**His**	0.91 ^a^	0.06	6.19	0.96 ^b^	0.05	5.21
**Lys**	2.14 ^a^	0.15	6.83	2.26 ^a^	0.12	5.31
Arg	2.58 ^a^	0.16	6.2	2.66 ^a^	0.15	5.64
Cys	0.52 ^a^	0.01	1.19	0.55 ^b^	0.02	3.64
**Met**	0.23 ^a^	0	1.25	0.23 ^a^	0.01	4.35
Total	33.27 ^a^	0.13	6.19	34.82 ^a^	0.12	5.57

Results are presented as mean values from three or more independent replicate experiments (n ≥ 3). Standard deviation (SD) and coefficient of variation (CV) are reported in separate columns. Different superscript letters (a–b) within the same row indicate statistically significant differences between varieties according to Welch’s *t*-test (*p* < 0.05). Essential amino acids are highlighted in bold.

**Table 2:** Free amino acid content in black soybeans and yellow soybeans in g/100 g dry matter.

	Black Soybeans			Yellow Soybeans		
Amino Acid	Mean	SD	CV%	Mean	SD	CV%
Asp	0.045 ^a^	0.000	1.00	0.041 ^a^	0.003	7.41
**Thr**	IC	IC	IC	0.015	0.003	21.67
Ser	0.029 ^a^	0.000	0.38	0.026 ^a^	0.001	5.66
Glu	0.101 ^a^	0.009	8.97	0.048 ^b^	0.004	8.99
Pro	0.004	0.000	4.15	IC	IC	IC
Gly	0.011 ^a^	0.000	4.20	0.003 ^b^	0.000	9.18
Ala	0.072 ^a^	0.002	3.42	0.011 ^b^	0.001	11.93
**Val**	0.005	0.000	1.26	IC	IC	IC
**Ile**	0.005 ^a^	0.001	9.94	0.003 ^b^	0.000	9.23
**Leu**	IC	IC	IC	0.002	0.001	24.35
Tyr	IC	IC	IC	IC	IC	IC
**Phe**	0.056	0.000	0.56	IC	IC	IC
**His**	0.031 ^a^	0.024	76.70	0.019 ^a^	0.004	18.99
**Lys**	0.009 ^a^	0.000	1.95	0.007 ^b^	0.000	0.24
Arg	0.191 ^a^	0.005	2.52	0.104 ^b^	0.001	1.06
**Total**	0.564 ^a^	0.004	9.59	0.279 ^b^	0.002	10.79

Results are presented as mean values from three or more independent replicate experiments (n ≥ 3). Standard deviation (SD) and coefficient of variation (CV) are reported in separate columns. Different superscript letters (a–b) within the same row indicate statistically significant differences between varieties according to Welch’s *t*-test (*p* < 0.05). Essential amino acids are highlighted in bold. IC—inconclusive results.

**Table 3:** Fat content and selected fatty acids in black and yellow soybean seeds.

	Black Soybeans			Yellow Soybeans		
	Mean	SD	CV%	Mean	SD	CV%
Fat [% *w*/*w*]	19.47	0.41	2.054	18.59	0.37	1.99
Saturated fatty acids [% of total FA]						
Myristic acid	0.35 ^a^	0.008	2.245	0.34 ^a^	0.111	32.796
Palmitic acid	13.44 ^a^	0.417	3.104	12.61 ^a^	0.011	0.084
Stearic acid	10.37 ^a^	0.088	0.852	8.46 ^b^	0.182	2.147
Arachidic acid	0.63 ^a^	0.035	5.585	0.66 ^a^	0.057	8.632
Behenic acid	0.4 ^a^	0.001	0.175	0.41 ^a^	0.033	7.895
Total	25.19 ^a^	-	-	22.48 ^b^	-	-
Unsaturated fatty acids [% of total FA]						
Oleic acid	25.44 ^a^	0.114	0.448	23.8 ^b^	0.164	0.689
Elaidic acid	0.95 ^a^	0.008	0.889	0.74 ^b^	0.068	9.161
Linoleic acid	42.14 ^a^	0.144	0.342	46.35 ^b^	0.133	0.287
Linolenic acid	6.13 ^a^	0.041	0.669	6.63 ^b^	0.050	0.693
Total	74.66 ^a^	-	-	77.52 ^b^	-	-

Results are presented as mean values from three or more independent replicate experiments (n ≥ 3). Standard deviation (SD) and coefficient of variation (CV) are reported in separate columns. Different superscript letters (a–b) within the same row indicate statistically significant differences between varieties according to Welch’s *t*-test (*p* < 0.05).

**Table 4:** Elemental composition of black soybeans and yellow soybeans in mg/kg.

	Black Soybeans			Yellow Soybeans		
Element	Mean	SD	CV%	Mean	SD	CV%
Ca	2978.8 ^a^	2.4	0.1	2833.4 ^b^	10.1	0.4
Fe	140.6 ^a^	1.5	1.0	130.2 ^b^	0.6	0.5
Mg	3008.1 ^a^	0.7	0.0	2911.1 ^b^	6.2	0.2
Mn	38.5 ^a^	0.2	0.6	40.7 ^b^	0.1	0.3
P	6153.2 ^a^	37.6	0.6	6136.5 ^a^	15.5	0.3
Zn	84.5 ^a^	0.6	0.7	91.4 ^b^	0.1	0.1
K	15,275.5 ^a^	69.4	0.5	16,436.7 ^b^	81.2	0.5
Na	92.8 ^a^	0.4	0.4	12.4 ^b^	0.1	1.1

Results are presented as mean values from three or more independent replicate experiments (n ≥ 3). Standard deviation (SD) and coefficient of variation (CV) are reported in separate columns. Different superscript letters (a–b) within the same row indicate statistically significant differences between varieties according to Welch’s *t*-test (*p* < 0.05).

**Table 5:** Trypsin inhibitor activity (TIU/mg) in black soybeans and yellow soybeans.

	Black Soybeans (TIU/mg)			Yellow Soybeans (TIU/mg)		
	Mean	SD	CV%	Mean	SD	CV%
TIA	30.22 ^a^	0.50	1.64	40.71 ^b^	0.47	1.14

Results are presented as mean values from three or more independent replicate experiments (n ≥ 3). Standard deviation (SD) and coefficient of variation (CV) are reported in separate columns. Different superscript letters (a–b) within the same row indicate statistically significant differences between varieties according to Welch’s *t*-test (*p* < 0.05).

## Data Availability

The data are contained within the article.
